# Before the wave: Exploring early perspectives on COVID-19 self-testing among African Americans in Eastern North Carolina

**DOI:** 10.1371/journal.pone.0330513

**Published:** 2025-09-03

**Authors:** Tiarney D. Ritchwood, Kelsey Burton, Mysha Wynn

**Affiliations:** 1 Wake Forest University School of Medicine, Winston-Salem, North Carolina, United States of America; 2 Duke University School of Medicine, Durham, North Carolina, United States of America; 3 Project Momentum, Rocky Mount, North Carolina, United States of America; Aga Khan University Hospital, PAKISTAN

## Abstract

**Introduction:**

In the first year of the COVID-19 pandemic, African Americans in rural communities faced disproportionate rates of hospitalizations and deaths. The emergence of the omicron variant further exacerbated these disparities, with African American adults approximately four times more likely to be hospitalized for COVID-19 compared to their White counterparts. To curb the transmission of the virus, public health professionals advocated COVID-19 mitigation strategies, including testing. However, barriers such as limited testing sites, long wait times, and privacy concerns hindered testing accessibility in rural areas. This study characterizes the early perceptions and acceptability of COVID-19 self-testing among African Americans in eastern North Carolina.

**Method:**

We conducted semi-structured, small group discussions in November 2021 with participants representing three age groups (youth, adults, and older adults) from Eastern North Carolina. Data were analyzed thematically using deductive and inductive approaches.

**Results:**

Findings indicate that, while self-testing was a novel concept for many participants, there was a high acceptability of COVID-19 self-testing. Barriers included low self-efficacy, concerns about test accuracy, and perceived costs. Participants emphasized the convenience and comfort of self-testing, recommending clear instructions, video demonstrations, and a toll-free number for help.

**Conclusions:**

These insights offer valuable guidance for improving emergency testing strategies and protocols in response to future viral outbreaks and pandemic threats. Clear, user-friendly instructions and video demonstrations can enhance the accessibility of self-testing kits. Moreover, addressing concerns related to cost and test accuracy is crucial for widespread adoption. Public health campaigns should prioritize affordability, user-friendliness, and community-specific needs to foster resilience and equity in healthcare responses.

The first year of the COVID-19 pandemic presented a multitude of challenges and uncertainties; during that time, members of historically marginalized and minoritized racial/ethnic groups faced disproportionately high rates of COVID-19-related hospitalizations and deaths [1,2]. The omicron variant, for example, saw African American adults being nearly four times more likely than their White counterparts to be hospitalized for COVID-19-related complications, a disparity higher than that of any other racial/ethnic group during the pandemic [3]. For African Americans in rural communities, such disparities were exacerbated by socio-structural factors [4], including higher poverty rates, limited employment opportunities, increased prevalence of service industry jobs requiring close contact with the public, inadequate access to healthcare facilities, and healthcare provider shortages [5–7]. In response to escalating risks, public health professionals urged communities to adopt COVID-19 mitigation strategies, including the three Ws (i.e., ***W***ashing hands, ***W***aiting 6 feet apart, and ***W***earing a mask) [8–10], immunizations, and testing [11]. With the role out of vaccines being delayed and uptake of immunizations being slow [12,13], public health professionals began to encourage individuals to engage in COVID-19 testing as a primary public health prevention strategy.

Early in the pandemic, facilities-based testing was the primary means for testing the public for the rapidly spreading virus. However, this proved challenging, particularly for those in rural communities due to limited transportation options to local testing sites and long travel distances to testing facilities [14,15]. One study cited additional barriers, including long wait times for tests and results, discomfort during nasal swabbing, and concerns about privacy at testing sites [16]. In this context, COVID-19 self-testing (CVDST) emerged as a promising strategy that could increase awareness of one’s COVID-19 status and potentially curb transmission rates. CVDST refers to the process by which individuals collect their own specimen, typically a nasal swab, and perform a diagnostic test for COVID-19 outside of a clinical setting, such as at home. Most CVDST kits are antigen-based tests, which detect specific proteins from the SARS-CoV-2 virus and provide results within 15–30 minutes. These tests are designed for ease of use and do not require laboratory processing. In contrast, PCR-based self-tests are less common and typically involve mailing the collected sample to a laboratory for analysis, with results returned in 1–3 days. CVDST is intended to increase accessibility to testing, reduce transmission, and support prompt isolation and treatment decisions. Specifically, CVDST was presented as a potential solution to barriers linked to facility-based testing, including offering increased privacy, convenience, elimination of distance-related challenges, and potentially improving the overall testing experience [17,18]. However, at the time of this study, self-testing was relatively new for much of the public, with few individuals having direct experiences with CVDST [17].

To guide our exploration of CVDST acceptability and feasibility, we drew upon the Health Belief Model (HBM) [19], which posits that individuals’ engagement in health-related behaviors is influenced by their perceived susceptibility to a condition, perceived severity of the condition, perceived benefits of taking action, perceived barriers to action, cues to action, and self-efficacy. This framework is particularly relevant for understanding how individuals assess the value and practicality of self-testing during a public health crisis, and it provides a useful lens for interpreting the facilitators and barriers identified in this study. Therefore, the purpose of this article is to highlight lessons learned and early perceptions surrounding CVDST during a critical juncture of the pandemic when the delta and omicron variants of COVID-19 were spreading quickly, and hospitalizations were on the rise. We sought to characterize the acceptability and feasibility of CVDST among African American adults in eastern North Carolina during the earlier stages of the pandemic. Insights from this research could inform future public health prevention strategies and testing campaigns, guiding efforts to increase self-testing in vulnerable communities facing emerging viral outbreaks or pandemic threats.

## Methods

In November 2021, we conducted semi-structured, small discussion groups with African Americans living in eastern North Carolina to identify barriers and facilitators to CVDST. Participants were recruited purposively and represented three age groups, including youth (ages 18–29 years), adults (ages 30–49 years), and older adults (ages 50 years and older). Groups were conducted in-person at a local public library between October and December 2021 and included 8–12 participants in each group. Each participant provided written informed consent, and two experienced members of the study team facilitated each group. This study was approved by institutional review boards at Wake Forest University School of Medicine and Duke University School of Medicine.

We developed an interview guide through an iterative process informed by existing literature on COVID-19 health behaviors and disparities. The guide was designed to explore participants’ perceptions regarding the impact of the COVID-19 pandemic on their daily lives, experiences with being diagnosed with or caring for someone with COVID-19, their engagement in COVID-19 prevention practices, including their testing-related experiences, as well as factors that either made it easier or more difficult to engage in COVID-19 testing. The guide was reviewed by our lead community partner for clarity, relevance, and flow and feedback was used to refine the questions and structure. Interviews were conducted in-person by 1–2 trained facilitators per group using a standardized protocol to ensure consistency across sessions. During the discussion groups, facilitators showed participants a short demonstration video depicting how to self-administer a COVID-19 self-test developed by a test manufacturer and publicly available online. We asked participants to share their opinions about self-testing and the kit being demoed, including their beliefs, motivations, and intentions regarding administering a COVID-19 self-test, and suggestions regarding product marketing and community-level interest in self-testing. We audio-recorded the small discussion groups, which were then transcribed by a third-party organization and reviewed for quality and accuracy by a research team.

Data were coded and analyzed using NVivo and deductive and inductive methods. Two researchers independently coded the first three transcripts and collaboratively developed a codebook, resolving discrepancies through discussion to achieve at least 95% inter-coder agreement. The remaining transcripts were coded by at least two members of the study team, with weekly meetings held to review coding consistency, refine the codebook, and ensure analytic depth. Specifically, we conducted codebook thematic analysis. This method involves developing a structured codebook that includes a list of codes, their definitions, and examples of how they are applied to the data [20,21]. Initially, codes were derived deductively from the interview guide and relevant literature. Additional codes were then developed inductively during the coding process to capture emerging themes [20,21]. The codebook was iteratively refined through team discussions to ensure clarity, consistency, and comprehensive coverage of the data. This approach supports transparency, strengthens inter-coder reliability, and facilitates collaborative analysis.

## Results

### Participants

Eighty-two individuals aged 18–90 years participated in the small discussion groups. The participants were categorized into three age groups: 15 young adults (18–29 years old), 29 adults (30–49 years old), and 38 older adults (50 years and older). About 75% of participants identified as female, and 62% were employed at the time of data collection. Participants lived in Nash County (48%), Edgecombe County (38%), and other areas in the eastern region of North Carolina. Notably, 78% of participants had undergone COVID-19 testing at least once though most had gone to testing facilities, as self-testing options were not widely available at that time. While preferences regarding the type of COVID-19 testing (e.g., self-testing vs. facility-based testing) varied, the primary focus of this study was to identify facilitators and barriers to the uptake of CVDST. We asked participants to share their experiences with COVID-19 testing before watching the CVDST demonstration video.

### Barriers for CVDST

Small discussion group participants identified three barriers to CVDST, including low testing self-efficacy, concerns about the accuracy of self-test results, and the potential cost of self-testing kits.

#### Low testing self-efficacy.

Several participants expressed low self-efficacy, or lack of confidence in their ability to follow kit directions and accurately perform the test. These participants expressed preferences for facilities-based testing, reporting fears associated with misinterpreting test results. As one participant from the study said,

*I’m afraid I might not get enough of what I’m supposed to get. I might not go far enough up [my nose]. The directions don’t say go into this region or that region, so I don’t want to do it wrong. -* Older Adult Small Discussion Group

When asked about their preferences for facilities-based testing over self-testing, some participants alluded to the importance of trust and skill, suggesting that professionals would be better able to administer the test than they, themselves, would. For example, a participant from the older adult small discussion group said, *“Because they know what they’re doing. I don’t know what I’m doing.”* Another participant from the Adult Small discussion group said, *“…I’m probably going to mess it up anyway…so I better go [to a facility] and let somebody professional do right.”*

Some participants attributed their low self-efficacy to their perception that the instructions seemed too complicated, with one person saying,

*Basically, you got too many steps to do it. You talk about taking this out. We have to measure how many drops you got to put in there. Then you have to unwrap the thing. Then you have to put that in there. I wouldn’t do it for the fear of missing a step*. – Older Adult Small Discussion Group

In the young adult small discussion groups, most participants reported finding the CVDST kits easy to use and were more comfortable with self-administration than other age groups. For those reporting discomfort with self-administration, concerns were like those of other age groups. One participant, for example, said, “I don’t think I would feel comfortable [using the self-testing kit], only because I know me. I’d feel like I’d do it wrong.”- Young Adult Small Discussion Group*.*

#### Accuracy of COVID-19 self-testing kits.

Some participants expressed concerns about kit accuracy, linking the kits’ ease of use to inaccuracy: “*[My question is, since] the process was so easy…is it accurate?”* During the demonstration, participants were notified that the CVDST kits may yield a false positive or false negative result. As a result, some participants had less interest and confidence in using them. Moreover, since self-test users are encouraged to confirm their results through facilities-based testing, the usefulness of self-testing was questioned: *“If you get a false positive with one of these [kits], you’d still have to get a follow up test. It would kind of seem like it’s pointless. What was my point of doing it?”* – Young Adult Small Discussion Group

#### CVDST kit cost.

The potential cost of CVDST kits was also cited as a barrier to uptake. As one participant shared in an adult small discussion group, “*If it all boils down to me paying for it, I’ll just let the providers provide it.” Another participant said*,

*…You get a test for free at urgent care…am I going to spend my money on a test that I don’t know if the results are going to come out [accurate] or not? - Adult Small discussion Group*

Since the cost of the kits were unknown at the time, participants shared concerns that using the self-test kits could be expensive and potentially wasteful, especially if results were inconclusive or provided a false positive. One participant explained the frustration that would likely accompany the mounting costs associated with verifying their COVID-19 test results,

*If it’s going to give me false readings, I’m going to be upset because I have spent my money on something that did not give me accurate information. So…what? Now I have to go out and buy another kit? Because some people on fixed incomes can’t afford to keep going out buying kits because of false positives and negatives. -* Older Adult Small Discussion Group

While more participants in the young adult small discussion group reported a willingness to purchase CVDST kits than those in other age groups, there was acknowledgement that the cost of the kits could be burdensome for people who were unemployed or had large families. When asked, ‘How do you think people in your age group would feel about self-testing?’, a participant from the young adult small discussion group said,

*… [there are more than two people in] a lot of families…so it’s like you will force them to buy more kits. So, then, it’s like now they’re looking at ‘do I want to buy more kits, or do I want to go to a facility where everybody can go for free?’* – Young Adult Small Discussion Group

### Facilitators of CVDST

We asked small discussion group participants to share their thoughts about things that made it easier for them to engage in CVDST. Most participants highlighted convenience and comfort as primary factors influencing their decisions.

#### Convenience.

Participants highlighted convenience as a key benefit to CVDST when compared to facility-based testing. Specifically, they believed that CVDST could address challenges typically associated with facility-based testing, including the need to travel long distances to reach testing sites and inadequate transportation options, particularly for those without personal vehicles or who were unable to drive themselves. Additionally, participants appreciated the fact that home-based CVDST enabled them to avoid the logistical burdens and safety concerns associated with in-person testing that were prevalent early in the pandemic, including delays in receiving one’s results and the need to have contact with others who might be infected. For example, an adult small discussion group participant commenting on the logistical burden said, *“If I have to make an appointment and its several days out, then I’d rather just go ahead and [do it] at home.”* Another participant said,

*You can do it, and then you don’t have to really wait. Most of them say you have to wait (for a long time) but with this one, you just take 10 minutes, and it is right there. -* Young Adult Small Discussion Group

A participant commenting on the potential of CVDST to prevent exposure to the virus in public settings said,

*[I prefer CVDST] *because you don’t have to be around a lot of people when you do it. You can sit there and do your own thing, and you don’t have to worry about waiting in line, a hundred people behind you, breathing on you. You can sit right there and do it by yourself**. – Older Adult Small Discussion Group

#### Comfort.

Participants also cited comfort as a facilitator to CVDST. Most participants with experience receiving a COVID-19 test at a facility reported nasal discomfort as a barrier to testing uptake. One participant in the older adult small discussion group shared their perceptions regarding why other members of their community were not getting tested for COVID-19 said, *“A lot of people are complaining about how far they ram it up their noses, which makes a lot of people want to push away because they don’t want to go through that.”* After viewing the CVDST demonstration video, participants in favor of self-testing reported that they would rather use the kit because it appeared painless and was preferrable to having someone else administering the test to them. As one participant said, “*I think [using the self-test] looks more comfortable, doing it on your own, because the way they do them at the health departments…[it’s] like they’re digging for gold.”-* Older Adult Small discussion Group

Participants highlighted the ability to control the testing wand played a part in helping to minimize the discomfort. An older adult participant said they preferred the self-test because, *“I know how much pressure is going up my nose.”* A young adult participant said:

*It sounds pretty easy because you can do it at your own pace at your own force. You don’t have to worry about somebody shoving it up your nose without even giving you a warning or anything.*

### Suggested resources

Participants provided suggestions on ways to increase the uptake of COVID-19 self-testing, highlighting the need for 1) video demonstrations and 2) a toll-free number for help and support. Participants recommended that kits include a video providing step-by-step instructions. For some, it was helpful to observe another person using a kit in their presence prior to self-administration, with a participant from an older adult small discussion group saying*, “the video can be helpful too… [to have] somebody actually showing you how to do it before you actually take it home and mess the whole kit up is helpful”.* A participant in a young adult small discussion group said, *“There should be some type of app with a video that has the same instructions on the app for somebody that just needed to visually see how to [use the self-test kit].”*

Participants also recommended a toll-free phone number be included with the self-testing kits. Participants thought it would be helpful to be able to speak with a health professional or other trained personnel to answer questions about the self-testing kits, help with test interpretation, and other COVID-19-related questions. According to one participant from an older adult small discussion group, “*You can always put something on the box, or some information be in there, where they can contact someone.”* A participant from an adult small discussion group said, “*I think they should have some type of nurse line that they can call and when they test positive, they can ask questions.”*

## Discussion

This study sought to explore community perceptions of CVDST among African Americans in rural North Carolina. Our goal was to provide insight on ways to improve the uptake of self-testing, particularly during a time in which information regarding COVID-19 was evolving, and self-testing was relatively new to the larger population. Our results suggested high acceptability of CVDST. Prior to the widespread availability of self-testing kits, preferences among participants varied, with some participants, especially older adults, reporting low self-efficacy largely due to concerns about their ability to follow the instructions and receive accurate results. Yet, most participants noted several benefits to using the kits, including convenience and privacy, and the absence of logistical challenges (e.g., the need for transportation, long wait times to receive a COVID-19 test, delays in receiving one’s results, and challenges scheduling an appointment at facilities-based testing sites). Our findings are consistent with those of previous studies, highlighting the usefulness of self-testing options in mitigating the impact of pandemic threats [22,23].

Participants noted several barriers to the uptake of CVDST, including low self-efficacy, concerns about test accuracy, and cost. Specifically, some participants, primarily older adults, expressed doubts about their ability to accurately follow the instructions provided within the self-testing kits. Multistep instructions, those with verbose descriptions, and those at higher reading levels, may be difficult for many participants to follow. As such, developers should be intentional about creating instructional materials that rely upon pictural graphics, use words parsimoniously, and are written at a third or fourth grade level. This is consistent with previous research suggesting that simplistic and straightforward instructions increase the accessibility of health-related information [22]. Participants also raised concerns about the accuracy of self-testing, questioning its usefulness given the potential for false positive or negative results. While such concerns are valid and relevant to various types of disease self-testing kits [24], it is important that public health professionals develop health communication campaigns that emphasize the potential benefits of self-testing kits. By addressing the practical challenges associated with facilities-based testing, such as transportation issues and lengthy wait times, self-testing presents as a convenient and accessible alternative. Additionally, culturally and community-tailored campaigns that suggest when these kits are most useful, such as when experiencing symptoms, before and after large gatherings, or following suspected and confirmed exposure) may help increase their uptake. Such strategies may increase self-testing uptake among community members, fostering a more proactive approach to managing the spread of infectious diseases.

Our findings are consistent with interpretations of well-known health behavior theories and models. The Health Belief Model (HBM), for example, posits that individuals are more likely to engage in health behaviors when they perceive themselves to be susceptible to a condition, believe the condition has serious consequences, and view the benefits of action as outweighing the barriers [19]. Research in the US as well as other countries, including China, have made similar assertions [25,26]. In our study, people more concered about CVDST barriers, such as cost and complexity of instructions, also shared beliefs consistent with low testing self-efficacy, while those more intereste in benefits, such as convenience and comfort shared beliefs consistent with high testing self-efficacy. As such, public health messaging emphasizing susceptibility, severity, and the benefits of self-testing, while anticipating and addressing potential barriers, may improve uptake.

Our findings also highlighted concerns about the potential cost of self-testing kits, with participants expressing reluctance to invest in self-testing kits, especially knowing that the results could be inconclusive or inaccurate. The perceived financial burden and the uncertainty of accurate results were key deterrents, particularly for individuals on fixed incomes. Some participants expressed a willingness to endure the logistical challenges associated with facilities-based testing because it was offered at no cost. Later in the pandemic (and after study completion), the government mailed free self-test kits to much of the country. While data on actual use of these kits are limited, many people requested the kits via online platforms. As the pandemic landscape continues to evolve, we face threats from both novel and reemerging viruses. These threats are exacerbated by politically motivated anti-vaccine rhetoric that undermines public health efforts, may contribute to conditions that enable future pandemics. In response, it is imperative that we identify strategies that ensure self-testing kits are both affordable, available, and widely used, and that the messaging on their appropriate use is clear. Such kits should be provided for free or at a low cost during the early stages of viral outbreaks to encourage widespread adoption and mitigate financial barriers.

Participants noted several facilitators of CVDST, including convenience and comfort. Regarding convenience, in addition to the elimination of travel and scheduling challenges associated with facility-based testing, participants cited the ability to perform tests in the privacy of one’s home thereby avoiding potential viral exposure and transmission, as a key facilitator to uptake. They also highlighted concerns about the discomfort associated with facility-based testing, with several reporting the pain of the procedure as off-putting. The perceived pain reduction, paired with the control participants felt over the testing process, contributed to a preference for self-administered tests. Relatedly, participants provided suggestions aimed at improving the uptake of CVDST. Clear and concise instructions are essential, with a particular emphasis on readability. Video demonstrations were recommended, underscoring the need for visual aids to supplement written instructions. Additionally, participants advocated for a toll-free number to get help, emphasizing the importance of accessible guidance from health professionals. From a diffusion of innovation perspective, CVDST can be viewed as a public health innovation that follows a predictable adoption curve, with early adopters being more open to new technologies and late adopters needing more support and reassurance [27]. Our findings suggest that younger participants were more likely to adopt CVDST early, while older adults expressed hesitancy. Tailoring outreach strategies to each adopter group, such as using peer testimonials for early adopters and offering hands-on demonstrations for late adopters, may accelerate adoption and reduce disparities.

Understanding the barriers and facilitators of disease self-testing, particularly during a viral outbreak, could help us with developing responsive public health interventions that consist of tailored educational materials that address low self-efficacy concerns, provide clear instructions and video demonstrations, and consider cost reduction strategies. Public health campaigns could emphasize the convenience and comfort of self-testing, addressing the barriers identified in this study. Moreover, the inclusion of a toll-free helpline can bridge information gaps for those without regular access to mass and social media and offer support, contributing to a more inclusive and accessible testing approach. Further, the lessons learned from our study can contribute to devising strategies for overcoming barriers to the uptake of CVDST if another novel pandemic threat emerges. Firstly, recognizing the financial constraints associated with self-testing kits, public health initiatives, such as those launched during the COVID-19 pandemic, should explore avenues for securing funding to make these kits available at little to no cost. This could involve collaborations with governmental bodies, private sectors, and philanthropic organizations to ensure the affordability of self-testing kits for the broader population. Moreover, enhancing the clarity and accessibility of instructions is also important. Designing user-friendly instructional materials at lower reading levels and incorporating video demonstrations can demystify the self-testing process, potentially boosting confidence and reducing the perceived complexity. Establishing toll-free helplines staffed by healthcare professionals or other trained personnel, as suggested by participants, could serve as a crucial resource for addressing concerns, clarifying doubts, and ensuring proper test interpretation, further overcoming potential barriers. The proactive integration of these strategies into public health preparedness plans can enhance community resilience and empower individuals to engage in self-testing during unpredictable viral threats, ultimately contributing to a more effective and equitable response.

While this study contributes valuable insights, it is not without limitations. Primarily, the assessment of self-test perceptions was geographically confined to communities in rural eastern North Carolina, potentially limiting the generalizability of findings to other areas of the state, as well as other regions in the US and the international community. Additionally, our study focused only on the perceptions and experiences of African Americans, which may limit generalizability to other ethnic/racial groups. Still, our findings were similar those from other studies focused on people living in the northeast and/or urban settings where a sizeable proportion of the sample identify as African/Black American [19]. Moreover, our sample offered valuable insights into the perceptions of CVDST within a distinct African American community context. A localized focus could not only improve our understanding of community-specific dynamics but could also lay the groundwork for future research to build upon this foundation and explore variations in perceptions across diverse regions. Another limitation pertained to our study’s focus on a self-testing kit that used a nasal swab rather than a saliva test. As such, the degree to which the knowledge and availability of other types of self-tests could affect willingness to test or engage is unknown. Additionally, the study relied on self-reported data, which may be subject to recall bias or social desirability bias. Participants may have over- or under-reported their comfort with or willingness to use self-testing kits given the group setting. Future research should consider triangulating self-reported data with behavioral data or follow-up interviews to validate findings. Finally, while our study captured early perceptions of CVDST, attitudes may have evolved as the pandemic progressed, and self-testing became more widely available. Longitudinal studies assessing how perceptions and behaviors changed over time and in response to public health interventions would have been greatly useful.

The results of this study offer actionable insights and lessons learned that can shape the trajectory of future self-testing campaigns not only for COVID-19 and other respiratory viruses, but also for conditions, such as HIV and other sexually transmitted infections. By addressing affordability concerns, emphasizing benefits, and considering community-specific needs, public health initiatives can better navigate the complexities of testing behaviors during public health emergencies. Additionally, our findings underscore the importance of tailoring self-testing strategies to address barriers such as low self-efficacy, perceived inaccuracy, and cost. Facilitators like convenience, comfort, and privacy should be emphasized in public health messaging. Integrating behavioral theories such as the Health Belief Model and Diffusion of Innovation Theory can guide the design of interventions that resonate with different segments of the population. As society continues to grapple with the aftermath and ongoing challenges of the COVID-19 pandemic, this work highlights the importance of preparedness efforts to prevent future pandemics. Public health professionals have an opportunity to leverage the lessons from this and other studies to mitigate barriers community members may encounter during emergencies or future pandemics, thus contributing to efforts to reduce health disparities and promote equitable access to testing.

## Supporting information

S1 Text CVDST Data Set. (DOCX)
