# Supplementary material for: Before the wave: Exploring early perspectives on COVID-19 self-testing among African Americans in Eastern North Carolina
Source: PLoS One. 2025 Sep 3;20(9):e0330513. doi: 10.1371/journal.pone.0330513 (PMC12407405; doi:10.1371/journal.pone.0330513)
Supplement: S1 Text — (DOCX) [file pone.0330513.s001.docx]

| **Theme** | **Sub-theme** | **Code-Subcode** | **Participant Quotes** |
| --- | --- | --- | --- |
| Barriers | Low testing self-efficacy | **COVID-19 Self-test perceptions- Reasons to NOT self-test** | *I’m afraid I might not get enough of what I’m supposed to get. I might not go far enough up [my nose]. The directions don’t say go into this region or that region, so I don’t want to do it wrong.”* (Older Adult Small discussion Group)  *“Because they know what they’re doing. I don’t know what I'm doing.”* (Older Adult Small discussion Group)  *“…I'm probably going to mess it up anyway…so I better go [to a facility] and let somebody professional do right.”* (Adult Small discussion Group)  *“Yes, a professional, that way I know it’s done correctly.”* (Adult Small discussion Group)  *“I mean, it might hurt or whatever, but at least they know what they're doing.”* (Young Adult Small discussion Group)  *I’d probably be scared I’m not pushing the swab back far enough or I push it back too far and I injure myself. (Adult Small discussion Group)* |
|  | Accuracy of COVID-19 self-testing Kits | **COVID-19 Self-test perceptions- Reasons to NOT self-test** | *“Cause the process was so easy, I think the steps were so easy. That would be my only thing is like if it’s accurate, accurate.”* (Adult Small discussion Group)  *“You know, so, the accuracy would be the ballgame. If its accurate, they can get my 10 dollars.”* (Adult Small discussion Group)  *“If you get a false positive with one of these [kits], you'd still have to get a follow up test. It would kind of seem like it's pointless. What was my point of doing it?”* (Young Adult Small discussion Group)  *“I wouldn’t feel like it would be accurate whether I did it, follow the steps completely. And for me, saying that is because I wouldn’t feel that the results would come back accurately because, I mean, you hear even whether you do it at home or go somewhere else, a lot of times those are not accurate.* You have *to do it again. So, I wouldn’t feel comfortable with buying a home kit and doing it at home.”* (Adult Small discussion Group)  *“I mean I would do it myself, but I’m just worried about being accurate or not. That, little lines and all that, that ain’t good enough for me.”* (Adult Small discussion Group)  *“I mean I would do it myself but I’d just be worried about the accuracy of it.”* (Adult Small discussion Group) |
|  | CVDST kit cost | **COVID-19 Self-test perceptions- Reasons to NOT self-test** | “*If it all boils down to me paying for it, I’ll just let the providers provide it.”* (Adult Small discussion Group)  *“…You get a test for free at urgent care…am I going to spend my money on a test that I don’t know if the results are going to come out [accurate] or not?”* (Adult Small discussion Group)  *“If it's going to give me false readings, I'm going to be upset because I have spent my money on something that did not give me accurate information. So…what? Now I have to go out and buy another kit? Because some people on fixed incomes can't afford to keep going out buying kits because of false positives and negatives.”* (Older Adult Small discussion Group)  *“… [there are more than two people in] a lot of families…so it's like you will force them to buy more kits. So, then, it's like now they're looking at ‘do I want to buy more kits, or do I want to go to a facility where everybody can go for free?”* (Young Adult Small discussion Group)  *“If the test costs, I’m gone go get a test [at the facility]”* (Adult Small discussion Group)  *“But some people aint got that twenty-three dollars.)* (Older Adult Small discussion Group)  *“That’s still high [the cost of the self-test kits] for people that just get paid once a month. No.”* (Adult Small discussion Group)  *They're not going to do. Yeah, they're young and it depends on the price.”* (Young Adult Small discussion Group)  *“I mean, because some people are not working still, so it just depends on the price as well.”* (Young Adult Small discussion Group)  *“So, I'm OK with it but I like the free ones. I can just go and to save time.”* (Young Adult Small discussion Group) |
| Facilitators | Convenience | **COVID-19 Self-test perceptions- Reasons to self-test** | *“If I have to make an appointment and its several days out, then I’d rather just go ahead and [do it] at home.”*  (Adult Small discussion Group)  *“You can do it, and then you don't have to really wait. Most of them say you have to wait (for a long time) but with this one, you just take 10 minutes, and it is right there.”* (Young Adult Small discussion Group)  *“[I prefer self-test] because you don’t have to be around a lot of people when you do it. You can sit there and do your own thing, and you don’t have to worry about waiting in line, a hundred people behind you, breathing on you. You can sit right there and do it by yourself*.” (Older Adult Small discussion Group)  *“I think I would [use the self-test] because I always tested at CVS and they did it in the car and they give you the kit and you do everything yourself. So, I feel like it will be the same thing I'll just get my results right then versus waiting a few days.”* (Adult Small discussion Group)  *“… you get to control it [self-test]. And you don't have to leave your job or your house and get exposed to COVID.”* (Young Adult Small discussion Group)  *“I think it depends on the scenario. If I am about to have a group of friends over and I just need something quick.” …If it's something like the airport would take it, then I’d spend a little bit more so, it just depends on the scenario.* (Young Adult Small discussion Group)  *“It's [self-test] convenient though..”* (Young Adult Small discussion Group)  *“So I'm OK with it but I like the free ones. I can just go and to save time.”* (Young Adult Small discussion Group)  *“For somebody like me, I work from 10:00 AM in the morning to like 8:30 PM at night, so I don't have time some days to go somewhere to get tested because everything is closed so that [self-test kits] would be way more helpful because I can just do the test.”* (Young Adult Small discussion Group) |
|  | Comfort | **COVID-19 Self-test perceptions- Reasons to self-test** | *“A lot of people are complaining about how far they ram it up their noses, which makes a lot of people want to push away because they don't want to go through that.”* (Older Adult Small discussion Group)  “*I think [using the self-test] looks more comfortable, doing it on your own, because the way they do them at the health departments…[it’s] like they’re digging for gold.”* (Older Adult Small discussion Group)  *“I know how much pressure is going up my nose.”* (Young Adult Small discussion Group)  *“It sounds pretty easy because you can do it at your own pace at your own force. You don't have to worry about somebody shoving up your nose without even giving you a warning or anything.”* (Young Adult Small discussion Group)  *“[Reason to self-test] I aint got to worry about somebody sticking it way up in my nose.”* (Older Adult Small discussion Group)  *I think I would feel comfortable [using the self-test kit] for the same reason I think they almost went to my brain one time, and they had my eyes watering then I went another time, and they say you don’t have to do it like that.* (Older Adult Small discussion Group)  *“Well like I said your nose is a very sensitive point of your senses and once someone touches it you just don’t feel completely ok with it but me I would do better with it by myself”* (Older Adult Small discussion Group)  *“Some nurses can be pretty rough they don’t realize how far they are putting it when I had mine, I didn't cry or nothing, but tears automatically came out of my eyes it’s just such a sensitive spot I was ready to slap her”* (Older Adult Small discussion Group) |
| Suggested resources | Help-Line | **COVID-19 Self-test perceptions- Resources needed to self-test** | *“Professional help, a help line with qualified professionals like she is claiming.”* (Older Adult Small discussion Group)  *“A telephone support person or somebody that can tell you or walk you through.”* (Older Adult Small discussion Group)  *“I think they should have some kind of number they can call to help navigate next steps.”* (Older Adult Small discussion Group)  *“And also, the kits should come with, even if you take it [self-test] or not, um a number.”* (Older Adult Small discussion Group)  *“Number you can call”* (Older Adult Small discussion Group)  *“I think they should have some type of nurse line that they can call.”* (Adult Small discussion Group)  “*You can always put something on the box, or some information be in there, where they can contact someone.”* (Older Adult Small discussion Group)  “*I think they should have some type of nurse line that they can call and when they test positive, they can ask questions.”* (Adult Small discussion Group)  *“To be able to have someone to talk to or even if it’s not no one to talk to, like have it on YouTube explaining to you each step and if it happens more than twice, what do we need to do.”* (Older Adult Small discussion Group) |
|  | Video |  | *“I would say for this new generation, you could have a QR code of some sort on the box where they can scan it and take you directly to a YouTube video or information that makes everything instant. It’s at their fingertips.”* (Older Adult Small discussion Group)  *“Play the video or something step by step”* (Older Adult Small discussion Group)  *“I mean the video can be helpful too, but along with somebody actually showing you how to do it before you actually take it home and mess the whole kit up just because you skipped one step.” (*Older Adult Small discussion Group)  *“There should be some type of app with a video that has the same instructions on the app for somebody that just needed to visually see how to [use the self-testing kit].”* (Young Adult Small discussion Group)  *“Just access to as much information as possible in as many different formats as possible. To make sure the test is being administered correctly.”* (Older Adult Small discussion Group)  *“To be able to have someone to talk to or even if it’s not no one to talk to, like have it on YouTube explaining to you each step and if it happens more than twice, what do we need to do.”* (Older Adult Small discussion Group) |
